# Supplementary material for: Optimization of differentiation and transcriptomic profile of THP-1 cells into macrophage by PMA
Source: PLoS One. 2023 Jul 17;18(7):e0286056. doi: 10.1371/journal.pone.0286056 (PMC10351730; doi:10.1371/journal.pone.0286056)
Supplement: S1 File — (ZIP) [file pone.0286056.s001.zip › supporting files/RNA-seq report.zip!/RNA-seq report/4_GOEnrichment_result/induce_vs_control/Enrich_bar/induce_vs_control.DEG_GO_Enriched_bar.pdf]

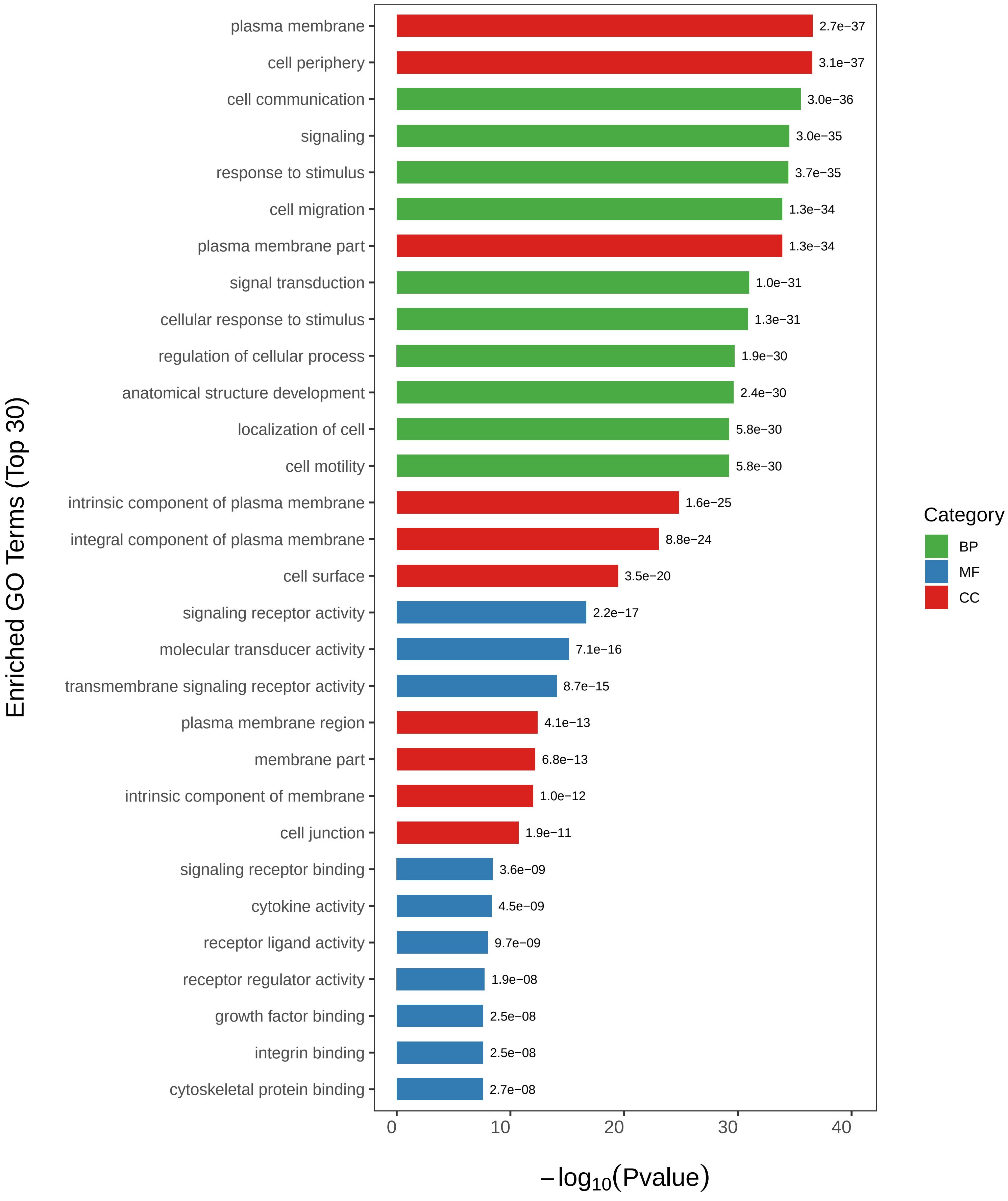

Enriched GO Terms (Top 30)
plasma membrane
cell periphery
cell communication
signaling
response to stimulus
cell migration
plasma membrane part
signal transduction
cellular response to stimulus
regulation of cellular process
anatomical structure development
localization of cell
cell motility
intrinsic component of plasma membrane
integral component of plasma membrane
cell surface
signaling receptor activity
molecular transducer activity
transmembrane signaling receptor activity
plasma membrane region
membrane part
intrinsic component of membrane
cell junction
signaling receptor binding
cytokine activity
receptor ligand activity
receptor regulator activity
growth factor binding
integrin binding
cytoskeletal protein binding
2.7e−37
3.1e−37
3.0e−36
3.0e−35
3.7e−35
1.3e−34
1.3e−34
1.0e−31
1.3e−31
1.9e−30
2.4e−30
5.8e−30
5.8e−30
1.6e−25
8.8e−24
3.5e−20
2.2e−17
7.1e−16
8.7e−15
4.1e−13
6.8e−13
1.0e−12
1.9e−11
3.6e−09
4.5e−09
9.7e−09
1.9e−08
2.5e−08
2.5e−08
2.7e−08
0
10
20
30
40
$-\log_{10}(\text{Pvalue})$
Category
BP
MF
CC
